# Change from Cardioinhibitory Syncope to Iatrogenic Positional Syncope: Superior Vena Cava Syndrome Treated by Superior Vena Cava Stenting and Leadless Pacemaker Implantation

**DOI:** 10.19102/icrm.2018.090902

**Published:** 2018-09-15

**Authors:** Firdevs A. Ekizler, Ozcan Ozeke, Riza S. Okten, Emek Edipoglu, Firat Ozcan, Serkan Cay, Serkan Topaloglu, Dursun Aras

**Affiliations:** ^1^Department of Cardiology, Health Sciences University, Turkiye Yuksek Ihtisas Training and Research Hospital, Ankara, Turkey; ^2^Department of Radiology, Health Sciences University, Turkiye Yuksek Ihtisas Training and Research Hospital, Ankara, Turkey

**Keywords:** Cardioinhibitory syncope, leadless pacemaker, positional syncope, superior vena cava stenting, superior vena cava syndrome

## Abstract

Symptomatic obstruction of the superior vena cava can be caused by either intrathoracic malignancy or nonmalignant etiology, resulting in superior vena cava syndrome (SVCS). The widespread use of central venous catheters, ports, pacemakers, and defibrillators has increased the incidence of benign SVCS. We present a post-pacemaker-implantation case of SVCS manifesting as positional syncope. The percutaneous intervention of stent implantation after lead removal followed by reimplantation of the leadless pacemaker may be a potential alternative treatment for pacemaker-induced SVCS, since some cases eventually may require repeat intervention.

## Introduction

Symptomatic obstruction of the superior vena cava (SVC) can be caused by either intrathoracic malignancy or nonmalignant etiology, resulting in SVC syndrome (SVCS). The widespread use of central venous catheters, ports, pacemakers, and defibrillators has increased the incidence of benign SVCS.^1^ Although pacemaker-induced SVCS is relatively benign, symptoms are often debilitating and refractory to drug therapy. Positional syncope has been well-described and is mostly neurocardiogenic in etiology; however, incomplete venous return secondary to SVC obstruction can result in a similar presentation.^2–4^

## Case presentation

A 39-year-old male presented to the clinic with a history of facial redness and swelling and recurrent syncope over the past year that was noticeably worse in the supine or bending forward positions or while doing push-ups/lifting weights. The patient had undergone DDDR [Talos DR, 60/60 beats per minute (bpm) with rate hysteresis of 10 bpm; Biotronik, Berlin, Germany] pacemaker implantation due to cardioinhibitory syncope that resulted in orthopedic injuries four years prior. His syncopal attacks resolved immediately after pacemaker implantation; however, they restarted with a change in nature at about one year later, occurring particularly in relation to him bending down to tie his shoelaces. Physical examination findings were unremarkable, except that, upon bending forward, the patient developed prominent venous collaterals on the neck and anterior chest/abdominal walls and positional syncope was reproduced. A computed tomography scan and venography confirmed SVC occlusion **(Video 1)** with a 14-mmHg gradient across the obstruction and total occlusion of the left subclavian vein **(Video 2)**. Following three months of oral warfarin therapy without any clinical benefit, the leads were separated from their middle part during mechanical traction from the subclavian vein and removed by use of a transfemoral snare system **(Videos 3 and 4)**. Subsequently, a balloon angioplasty was performed at a pressure of 8 atm.

**Figure 1:**
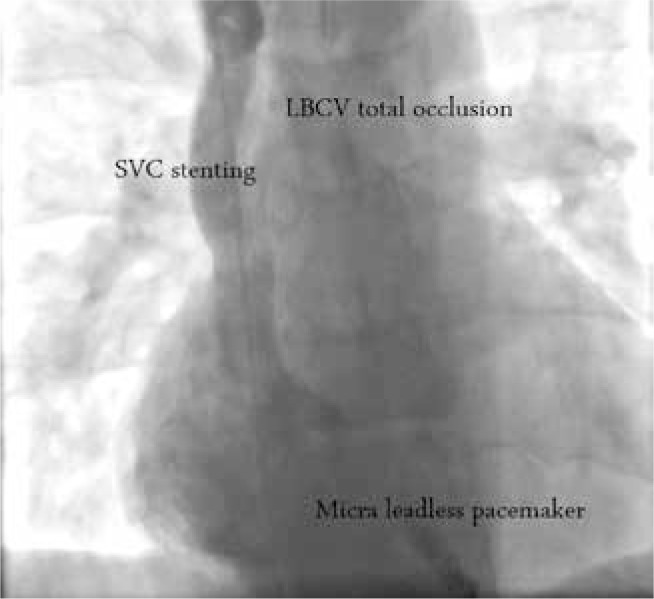
The SVC angiogram shows total occlusion of the left brachiocephalic vein, SVC stenting, and right ventricular apical Micra™ leadless pacemaker (Medtronic, Minneapolis, MN, USA) placement. SVC: superior vena cava; LBCV: left brachiocephalic vein.

Given the significant venous recoil, we decided to implant a stent. Using fluoroscopy, we confirmed placement in the proper stent position **(Video 5)** and finalized stent deployment by inflating the outer balloon to a pressure of 6 atm **(Figure 1** and **Video 6)**. Follow-up venogram results indicated no collateral flow, and the pressure gradient was found to be reduced from 14 mmHg to 1 mmHg. The patient had no postoperative complications and was discharged from the hospital the next morning, with instructions to take dual antiplatelet agents. Clinical follow-up after one month revealed marked improvement in his symptoms, including resolution of his positional syncope; however, his cardioinhibitory syncopal attacks had resumed. Therefore, two months later, we implanted a Micra™ leadless pacemaker (Medtronic, Minneapolis, MN, USA) **(Figure 1** and **Video 7**. At one year postimplantation, the patient had no syncopal events.

## Discussion

Although approximately 30% of patients receiving transvenous permanent pacemaker implants may have peripheral or central venous occlusion, only three per 10,000 implants to four per 1,000 implants will develop SVCS.^5^ The pathogenesis of SVCS is unclear. In patients with early presentation, acute thrombosis is the general cause, while fibrotic stenosis plays a role in chronic cases. In our case, fibrotic stricture was believed to be the cause of SVCS, as a computed tomography scan showed no definite hypodense material in the SVC and the patient’s symptoms persisted after anticoagulation. Endovascular management is the first-line treatment for SVCS caused by intravenous devices, while surgery is most often performed in cases of mediastinal fibrosis.^1^

In terms of technical application, the use of pressure measurements in the venous system remains under debate. A consensus has been established that a pressure difference (gradient) of 2 mmHg to 3 mmHg is significant in the venous system. However, in practice, instrument inaccuracy, respiratory variation, and transducer positioning among patients produce errors that exceed 2 mmHg to 3 mmHg. Therefore, the patient’s symptoms and degree of stenosis, rather than hemodynamics, should guide treatment. Any symptomatic venous narrowing can be considered to be an indication for venoplasty and venous stenting in lead-induced SVCS. In this case, various brands of angioplasty balloons were used at the discretion of the interventional radiologist, including balloons allowing medium- (8–12 atm) or high-pressure (18 atm) inflations. Since the SVC is surrounded by rigid structures (ie, the mediastinum, sternum, and right mainstem bronchus), it is more prone to obstruction relative to its neighbors. The performance of venous angioplasty by itself is usually not sufficient to keep a vein open, presumably because of the low intravascular blood pressure (as compared with that of the arterial system); therefore, metal stents are often required for long-term patency in venous disease.^6^ The diameter of the chosen stent is based on the diameter of the normal vein adjacent to the lesion. Larger stents perform better than smaller ones in venous procedures due to recoil effect; specifically, consideration should be given to oversizing by 10% to 20%. In practice, symptomatic SVC or inferior vena cava stenosis rarely require stents of more than 18 mm in diameter. The stents used in the venous system should be self-expanding in most cases. Balloon-expandable stents are only used when greater radial force is necessary and should not be employed in superficial areas. These stents are poor choices for implantation outside of the chest cavity or abdominal cavity. Good inflow is a key requirement for successful venoplasty and stent placement. Currently, there is no clear consensus regarding which pharmacologic agent is best for use after stenting, with various agents such as warfarin, aspirin, and clopidogrel having been tried for durations ranging from six months to the patient’s lifespan. Anticoagulation is not usually required following upper extremity stent placement unless patency is compromised by poor inflow.

Reintervention with percutaneous balloon venoplasty is successful in most patients with symptom recurrence.^7^ However, the use of a leadless pacemaker to treat neurocardiogenic syncope, while uncommon, has been increasingly reported in the literature.^8,9^ Furthermore, although successful results have been shared with respect to for stenting of the SVC with the leads in place,^10^ concerns persist with regard to the potential risk of lead damage and ensuing dysfunction caused by the metallic mesh of the stent. In addition, entrapment of pacemaker leads by a stent would make potential future extraction of the leads (eg, for infection) virtually impossible without a cardiac surgical procedure.^7^ The implantation of a leadless pacemaker eliminates important sources of complications associated with traditional pacing systems, such as lead failure, pocket complications, and infection, while providing similar pacing performance and potentially better psychological and aesthetic results.^9^ As such, the leadless pacemaker may be an effective alternative method in the treatment of recurrent neurocardiogenic syncope.
